# Non-verbal cognitive development, learning, and symptoms of PTSD in 3- to 6-year-old refugee children

**DOI:** 10.1007/s00431-021-04312-8

**Published:** 2021-11-24

**Authors:** Andrea Hahnefeld, Thorsten Sukale, Elena Weigand, Verena Dudek, Katharina Münch, Sigrid Aberl, Lea V. Eckler, Ina Nehring, Anna Friedmann, Paul L. Plener, Jörg M. Fegert, Volker Mall

**Affiliations:** 1 Chair of Social Pediatrics, TUM School of Medicine, Technical University of Munich, Munich, Germany; 2 kbo Kinderzentrum, Heiglhofstrasse 65, 81377 Munich, Germany; 3 Department of Child and Adolescent Psychiatry and Psychotherapy, University of Ulm, Ulm, Germany; 4 Department of Psychosomatic Medicine and Psychotherapy, Technical University of Munich, Munich, Germany; 5 Department of Child and Adolescent Psychosomatic Medicine, Munich Municipal Hospital Group, Munich, Germany; 6 Department of Child and Adolescent Psychiatry, Medical University of Vienna, Vienna, Austria

**Keywords:** Refugee, Children, Non-verbal IQ, Learning, PTSD, KABC-II

## Abstract

As IQ tests are commonly used as key assessment method, we address the question whether our commonly used standardized IQ tests are appropriate for children from families of diverse cultures and different educational levels in a refugee population. We examined 109 refugee children aged 3–7 years (*M* = 5.10 years, *SD* = 1.25) with the “Kaufman Assessment Battery for Children “ (KABC-II; Kaufmann & Kaufmann, 2015) on a language-free scale (Scale of Intellectual Functioning, SIF) and learning performance (subtest Atlantis). With a non-verbal IQ of 81.5 (*SD* = 18.01), the population mean of the refugee children is more than one standard deviation lower than the mean of the German norm population. Standardized scores follow the normal distribution and are not correlated to any of the assessed markers of adversity (flight duration, time spent in Germany, child PTSD in parent rating, parental symptom load, and parental education level).

*Conclusion*: The interpretation of IQ test results for refugee children should be done cautiously as results may underestimate their cognitive capacity. Environmental factors, such as high illiteracy among parents in this study, the lack of institutional education of children and high lifetime stress, may explain our findings.

*Trial registration*: DRKS00021150.

**Table:** 

**What is Known:** *• There is a high pervasiveness for the use of standardized IQ tests in the German health and education system to determine eligibility for special education and social services.*	
**What is New:** *• Refugee children score significantly lower than German children in a language-free IQ test. As results are normally distributed and not correlated to any of the assessed markers of adversity, the low scores in the refugee group might be due to missing formal education.*

**What is Known:**

*• There is a high pervasiveness for the use of standardized IQ tests in the German health and education system to determine eligibility for special education and social services.*

**What is New:**

*• Refugee children score significantly lower than German children in a language-free IQ test. As results are normally distributed and not correlated to any of the assessed markers of adversity, the low scores in the refugee group might be due to missing formal education.*

## Introduction

From 2015 to 2018, there have been 1.5 million people applying for asylum in Germany, among them about 18% children aged 6 years or younger [1]. Consequently, there has been growth in the number of families and children from culturally and linguistically diverse backgrounds seeking help in the German health system and needing to be placed in the educational environment [2]. Previous research shows that refugee children in Europe have health risks and special needs that might differ from peers in the local population [3–5]. For all children, a common right for education and schooling is declared in the Human Rights Convention [6]. If required, placement in educational settings in Germany is decided by children’s results in standardized IQ tests in combination with clinical observations [7], as intelligence does not only correlate with health outcomes [8] but also with school grades [9] and is considered to be a strong predictor of school achievement [10]. There is also a high pervasiveness in the use of standardized IQ scores to identify intellectual giftedness (IQ > 130) and mental retardation (IQ < 70) in a clinical context. Especially the latter is important in determining eligibility for special education and social services [11].

Yet there are good reasons to use caution in interpretation of test results for children with diverse ethnic backgrounds, as we know from previous research that children from families with migration background score lower (not only) in verbal domains [12]. Even when tests are administered in children’s dominant languages, lower scores encompassing 0.5 to 1.5 standard deviations have been reported for migrant children (where both parents have a migration background) as young as kindergarten age [13, 14]. Furthermore, in Lüdeke et al.’s [14] large sample of children with different developmental and psychiatric disorders, there was a significant effect for migration background, but no significant interaction with or effects for psychiatric disorders. These results suggest migration background as an important variable to explain limited performance in intelligence tests, and there might be a risk of classification and stigmatization based on these assessments.

Considering refugee children, who have often been exposed to an unpredictable environment with all corresponding risk factors [15], there is another line of evidence linking higher stress levels in combination with adversity and high arousal symptoms to poorer cognitive development in the long term [16, 17]. This raises the question whether the use of non-verbal neuropsychologically developed tests commonly used among German, Western European, and American children is appropriate and ecologically valid for populations of refugee children from diverse cultures and different educational levels, especially when used as a tool for school placement and decisions.

The following hypotheses were tested:

1. We expected our sample of refugee children to score lower than children from the norming population on the neuropsychologically developed language-free “Scale of Intellectual Functioning” (SIF) and in a visual learning task (subtest “Atlantis”) of the “Kaufman Assessment Battery for Children” (KABC-II) [18, 19].
2. We expected children with a higher trauma-specific symptom load and/or highly stressed parents to score lower than children with less trauma-specific symptoms and/or less stressed parents.

## Method

### Participants

The study was conducted between July 2018 and July 2021. The research was approved by the ethics committee of the Medical Faculty of the Technical University of Munich (221-18 s, 19.06.18).

Participants were recruited from five refugee camps. The study included children aged 3 to 7 years. All were accompanied by at least one parent. Families eligible for our study were contacted by the consultant team, received information about the study, and were invited to the first appointment, with interpreters if necessary. Children with a reported history of neurological conditions, obvious developmental delays or significant impairments were excluded. Overall, data of 109 patients with a mean age of 5.10 years (*SD* 1.25, range 2.92–7.92) were included in the study. Fifty of these children (mean age 5.2 years, *SD* 1.15, range 3.00–7.67) were also participants of the study “Survival states as indicators of learning performance and biological stress in refugee children: a cross- sectional study with a comparison group.” [20].

### Procedure

Written informed consent was given by the parents, and children provided verbal consent. Parents were asked for general information on age, sex, language, and cultural background. Formal schooling, duration of flight, and time since arrival in Germany were also assessed.

While children were examined with the SIF of the KABC-II [18] and the learning task Atlantis, parents filled out questionnaires in their native language (“PORTA”) [21]. As the majority had difficulties with the written format, the questionnaires were often administered as interviews with interpreters. Full descriptions of the procedure can be found in Hahnefeld et al. [20].

### Assessment

#### Measures

The KABC-II SIF has been developed as a language-free and culture-fair diagnostic instrument based upon Luria’s [22] neuropsychological framework and the Cattell-Horn-Carroll hierarchically organized model [23] with a norm population of more than 3000 children in the German version. The authors of the German version report no significant effects of sex on test scores. For the age group of 3- to 6-years-old children, there are moderate effects for parents’ level of education and migration background. These effects are stronger for children 7 years and older with children from higher educated parents and without migration background scoring higher. It has to be noted that children with low parental education are underrepresented in the norming sample [18, 24]. The KABC-II SIF has good psychometric properties concerning construct validity (correlations of *r* = 0.59 to *r* = 0.72 with other non-verbal test batteries) and reliability (split-half *r* = 0.90). It is considered culture-fair because only small score differences between ethnic groups are marked [18, 24]. We decided to apply the KABC-II because of (i) the comprehensive norming population of 519 children in the age group of our study (3–7 years) [18, 24]; (ii) the inclusion of a comparative sample from a different culture in the pre-norming phase (46 randomly selected Taos Pueblo children in New Mexico scored within the average range compared to age-matched American children on all the information-processing subtests and scales) [25]; and (iii) additionally, the test material proved to be appealing for younger children.

These three subtests of the SIF that were administered in all age groups :

- Concept Formation (CF): On templates with four to six pictures, the child is asked to choose the picture which does not fit in. This language-free subtest assesses conceptual thinking and the ability for visualization and inductive thinking in a multiple-choice format.
- Triangles (T): The child is supposed to copy/rebuild abstract representations with small building blocks as demonstrated by the examiner. The subtest assesses visualization and spatial thinking in an interactive way.
- Hand Movements (HM): The child is demanded to repeat the investigator’s hand movements. This task assesses sequential processing, seriation, and short-term memory in the visual-motor modality.

Additionally, the subtest *Atlantis* was conducted to assess the children’s short-term learning performance.

- It demands the children to memorize non-sense names of fishes, plants, and shells in illustrations of cartoon-like pictures.

The subtest Atlantis shows low intercorrelations with the general cognitive scale (*r* = 0.28) and is reported to be reliable (*r* = 0.97) and independent from the parents’ migration status [18].

With low correlations with tests for short-term selective attention (*r* = −0.06 SIF, *r* = −0.02 Atlantis), both tasks show good discriminant validity [18]. We assessed parental well-being with the “Refugee Health Screener” (RHS-15; Pathways to wellness) [26], by screening for emotional distress as a common marker across psychiatric diagnoses in many ethnic groups. The RHS is an empirically developed standard instrument and we followed the authors’ recommendation that “a score of ≥ 12 or a distress thermometer score of > 5 is considered a positive case” (p. 6) [26]. We calculated a sum score by multiplying the symptom score with the thermometer score.

All children were screened in parent rating for potentially traumatic experiences and symptoms of PTSD with the “Child and Adolescent Trauma Screening” (CATS) [27], a short open-accessible screening instrument directly based on the DSM-5 criteria for PTSD [28] with satisfactory psychometric properties, constructed with emphasis on sensitivity. We used the recommended cutoff ≥ 16 (total symptom score) as an indicator of a clinically relevant level of symptoms in preschool children.

#### Psychometric testing and statistical analyses

We did descriptive analyses for demographic data and conducted Shapiro–Wilk tests for normal distribution, and *t*-tests for independent samples and correlations (Pearson for normally distributed variables and Spearman for non-parametric testing). All calculations were done with IBM SPSS Statistics version 25.

## Results

### Participants

Of initially 127 eligible children with refugee background, complete IQ data could be obtained for 104 children (59% boys, 41% girls) from 89 families. Participants were from 16 nations, mostly of Afghan (50), Nigerian (23), and Syrian (13) origin. The mean duration of time since arrival in Germany was 16.5 months (*SD* 19.41, range 0.5–96 months) after an average flight duration of 24.5 months (*SD* 27.89, range 0–140 months). See Table 1 for description of the sample.

**Table 1:** Description of the sample

	*Mean*	*SD*	*Range*
Mean age (years)	5.10	1.25	2.92–7.92
Flight duration (months)	24.84	27.89	0–140
Time spent in Germany (months)	16.50	19.42	0.5–96
Non-verbal IQ: SIF from KABC-II (scale value)	81.50	18.01	44–142
Learning: subtest Atlantis (scale value)	6.79	3.01	1–15
CATS (symptoms of child in parent rating)	10.82	12.41	0–43
RHS (number of symptoms, parent self-rating)	22.01	15.79	0–55

### Test scores

**Fig. 1:**
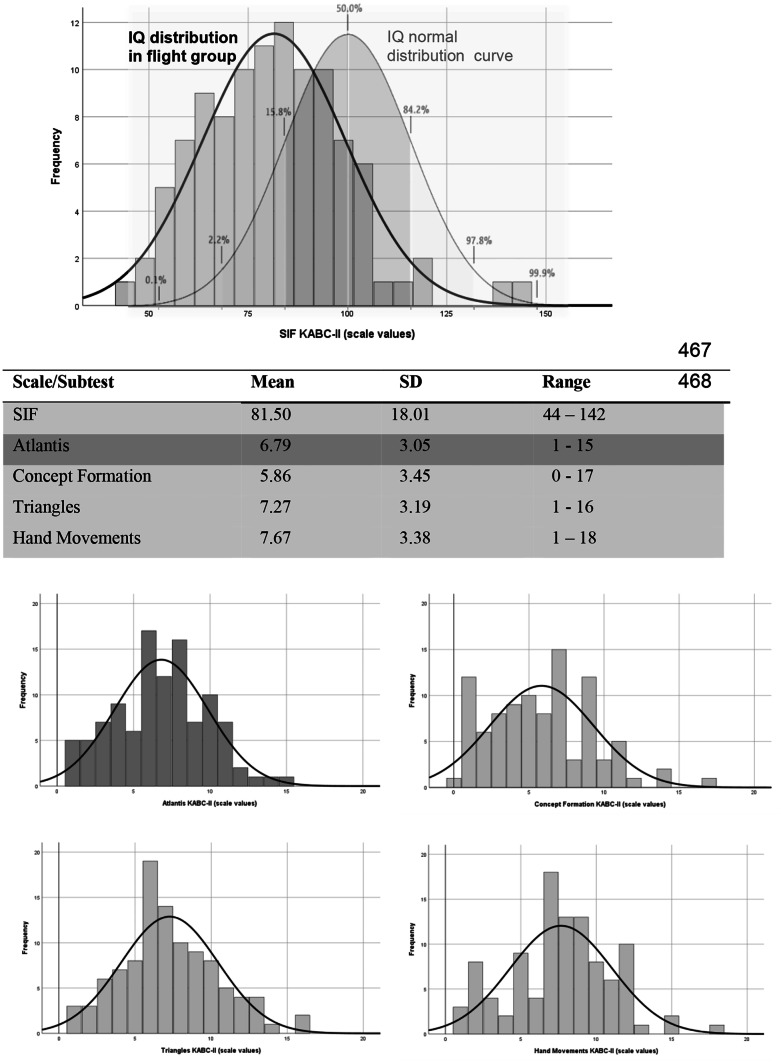
Histograms for S1F and subtests Atlantis, Concept Formation, Triangles, and Hand Movements (KABC-11, in scale values)

As represented in Fig. 1 and assessed by the Shapiro–Wilk test, distributions for scale values of SIF and subtests Atlantis, Triangles, and Hand Movements showed a normal distribution (*p* > 0.05, for SIF, Triangles, Hand Movements, and Atlantis).

For subtest Concept Formation, the scale values deviated from normal distribution (*p* < 0.05, Shapiro–Wilk test). For this subtest, we found floor effects with the lowest mean scale value among all subtests. In the clinical observations, we noticed that even with interpreters, a lot of children did not know what to do in this subtest. With an average non-verbal IQ (KABC-SIF) of 81.5 in our sample, we found significantly lower IQ scores for these refugee children compared to the population mean value of IQ = 100 (*t*_*SIF*_ (103) = −10.5, *p* < 0.001, two-tailed) with an effect size of *d*_*Cohen*_ = 1.396 equalling a large effect [29]. In total, on the non-verbal scale SIF, 26 children (24%) were below cutoff of IQ < 70 for mental retardation, 39 children (36%) were in the average range of IQ 85 to 115 and two children (2%) scored above average (IQ > 115). The children in our sample also displayed a significantly lower learning performance compared to the normative sample (*t*_*Atlantis*_ (105) = −10.75, *p* < 0.001).

Whereas parents reported potentially traumatic experiences for 67% of the children, 34% showed a clinically relevant level of symptoms in parent-rated CATS, and 26% fulfilled diagnostic criteria for PTSD according to criteria of DSM-5 in the screening. But 79% of the parents reported a symptom load above clinical cutoff for themselves. In our rough estimate of parents’ educational level, we found a 34% rate of illiterates. About 33% of the mothers never went to school, 46% had some years of education (varying from 2 to 10 years) without degree, about 9% reported a degree, and roughly 8% had enrolled in university training. For 4%, we did not obtain any reliable information on that topic. As we had a lot of single mothers in the study, we report only the mothers’ education level.

There were no significant correlations for IQ (KABC-SIF) and learning performance (subtest Atlantis) with children’s flight duration, child PTSD in parent-rated trauma screening (CATS), parental symptom load (RHS), and parental education level (rough estimate, self-rating) as summarized in Table 2. We did not find any significant differences in IQ when comparing children from different home countries (*F*_country_ (3) = 0.802, *p* = 0.495). Whereas there was no correlation for IQ with time spent in Germany, we found a significantly positive correlation with learning performance and time spent in Germany.

**Table 2:** Correlation coefficients (Pearson for age and Spearman rank correlations for other variables, two-tailed) for IQ (SIF KABC-II) and learning performance (subtest Atlantis) with flight duration, time spent in Germany, PTSD in parent-rated trauma screening (CATS), parental symptom load (RHS), and parental education level (*p < .05, **p < .001)

	IQ	Learning performance
	(SIF)	(Subtest Atlantis)
Flight duration	− .02	− .08
Time in Germany	.12	.27**
Child PTSD (CATS in parent rating)	.02	− 02
Parental symptom load (RHS self-rating)	.10	.01
Parental education (self-rating)	.01	− .07
Learning performance (subtest Atlantis)	.39**	1

## Discussion

In the Scale of Intellectual Functioning (SIF) of the KABC-II, we found normally distributed but significantly lower IQ and learning scores for refugee children compared to the population mean of German children. With a non-verbal IQ of 81.5, the population mean of our sample is more than one standard deviation lower than the mean of the German norm population. When applying the diagnostic criteria for mental retardation according to ICD-10-criteria of IQ < 70 [30], 24% of the children of our cohort would be classified in this category, compared to 3% in the norming population.

One reason for an increased rate of children with mental retardation among refugee children could be the negative effect of insecure environment on health and development, which has been repeatedly described [3, 15]. Furthermore, children with (cognitive) disabilities probably experience more social rejection and impairment in countries with political instability, and their families might therefore leave their country to search better medical support and treatment. However, the assumption of an increased percentage of mental disability in our sample is unlikely for several reasons: First of all, we excluded children with a reported history of neurological conditions, obvious developmental delays, or any known significant impairments from the study. Secondly, we observed largely age-adequate and clinically normal behavior for all children, and for none of the children, “deficits in two or more adaptive behaviors that affect everyday/general living” were reported as required for full diagnostic criteria for mental retardation in ICD-10 [30]. As a third argument, with a significant influence of children with mental retardation on the mean value of a population, we would expect a left-skewed dissymmetrical distribution, which is not corresponding to the normal distribution curve of our data. Consecutively, other factors with a high interindividual variability such as flight duration, time spent in Germany, trauma screening in parent rating, parental symptom load, and parental education level did not show any significant correlations with the IQ scores. Thus, our hypothesis that children with a higher trauma-specific symptom load and/or more stressed parents score lower than children with less trauma-specific symptoms and/or less stressed parents could not be confirmed. Nevertheless, we found a positive correlation between time spent in Germany and the children’s learning scores, which might point to the fact that a safe environment enhances learning performance.

By assuming migration or refugee background as a variable to explain limited performance in intelligence tests, several risk factors (e.g., exposure to violence, altered stress physiology, and high risk of mental health problems) are discussed in the sense of patterns that are shared for children on the move across regions of origin [3, 31]. Although we did not find a correlation of flight duration and trauma screening in parent rating, we are not able to exclude these effects for several reasons: (i) parents’ rating of child PTSD may not be sensitive to evaluate individual trauma experiences and dispositions as we have discussed before [20], (ii) several risk factors might be adding up for every child in an individual way.

Alternatively, there could be one common factor affecting our whole study population in a comparable way and accounting for the effect of a downward-shifted normal distribution for the IQ scores, such as educational experiences. As we had a 34% rate of illiterate mothers, we might have a sample with a very low education level in general. Lower educational levels are underrepresented in the norming population of developmental tests [24], which might to some part explain the lower performance of our sample. Furthermore, comparing individuals within the same culture with differing levels of formal education, Rosselli and Ardila [32] found a significant effect of educational level on children’s and adults’ performance in non-verbal neuropsychological tests, suggesting that environmental variables are strongly connected to the development of non-verbal executive functions in children which in turn influence test performance. Therefore, both lower educational level of parents and the lack of formal education of the children might have contributed to our findings.

Another factor might be effects of cultural bias. In our sample, refugee children from mostly Eastern/African countries were confronted with a test that was developed and normed in Western cultures, where children—in contrast to our study cohort—are usually exposed to books and pictorial materials equal to the test materials from early ages on and are often introduced to formal education by kindergarten age. Although the KABC-II has been studied in a group of Taos Pueblo children in northern New Mexico, there is an underrepresentation of children with non-western migration background within the normative sample [18, 24].

Furthermore, the children in the Taos study, although coming from a different cultural background (Indian vs. North-American), went to school and therefore had formal institutional education — unlike most of the participants in our study.

In fact, a closer look at the results of the subtests points towards this additional educational and cultural factor leading to bias when testing children with refugee background. We found the subtest Atlantis for learning behavior and the subtests Triangles and Hand Movements of the SIF to show face validity and normal distribution in our sample, whereas the results for subtest Concept Formation showed floor effects, no normal distribution, and less face validity. Many children obviously did not know what to do when being confronted with that task, e.g., kept naming all the pictures instead of pointing at the one out of four pictures that was from a different category. Corresponding observations with this task for logical reasoning have been described by Voigt [33]. Accordingly, Greenfield [34] recommends to avoid multiple-choice formats in populations without considerable formal education. The procedure of creating personal rapport between investigator and child by acting and turn-taking in the subtests Triangles and Hand Movements might be superior in assessing performance and knowledge to the procedure of demanding to show the right solution on a sheet of paper, especially for children from collectivistic cultures and no experiences with formal education [34].

Correspondingly, some authors have expressed a need for tests that are more relevant to the everyday requirements for populations in non-western countries [8, 13] with the remaining question whether changes in the tests themselves would make them more valid in other settings [31], or whether all that is needed are different normative standards [31, 35]. With the demand to memorize pictorial material, we rate the subtest Atlantis to show high relevance for everyday life. So in addition to obvious language barriers, there are several potential biases in testing children with minority status, migration, or flight background [13], and great caution is required when non-verbal neuropsychological tests are administered to individuals from cultures different from the one that provided the normative sample [32], particularly when using tests to support placement in educational settings [13] and to determine further services in clinical settings.

Suggestions for further research would be to perform longitudinal and intervention studies to investigate whether these abilities are sensitive to treatment and early education, especially for children from high-stress and low-home learning environmental contexts, and to what extent these children can catch up once they are in a safe environment with access to early education.

## Conclusion

Our study with a respectable sample size reflecting the diversity of refugee families coming to Germany demonstrates that IQ values as revealed by the KABC-II have to be interpreted cautiously as they might severely underestimate the cognitive capacity of these children. Environmental factors, such as high illiteracy among parents in this study, the lack of institutional education of the children, and high lifetime stress might be responsible for these findings.

## Data Availability

On reasonable request, all data and codes are available from the corresponding author.

## Abbreviations

CATS: Child and adolescent trauma screen
ICD-10: International classification of diseases
IQ: Intelligence quotient
KABC-II: Kaufman Assessment Battery for Children
PTSD: Posttraumatic stress disorder
RHS: Refugee health screener
SIF: Scale of intellectual functioning

## Declarations

### Disclaimer

No content of the manuscript has been written or edited by anyone other than those individuals listed as an author on the submission. The corresponding author Andrea Hahnefeld confirms that any tables and figures submitted with the manuscript are original and that the author is the copyright holder.

## References

[1] BAMF (2020) Aktuelle Zahlen zu Asyl, Ausgabe: Januar 2018. https;//www.BAMF.de. Accessed 23 Nov 2020

[2] ISSOP Migration Working Group (2020) ISSOP position statement on migrant child health. Child: Care Health Dev 44(1):161–170. 10.1111/cch.12485 10.1111/cch.12485 28736840

[3] Kadir A, Battersby A, Spencer N (2019). Children on the move in Europe: a narrative review of the evidence on the health risks, health needs and health policy for asylum seeking, refugee and undocumented children. BMJ Paediatrics Open.

[4] Nehring I, Schlag E, Qirjako E et al (2019) Health state of Syrian children and their parents in a German refugee camp, Journal of Refugee Studies fez029. 10.1093/jrs/fez029

[5] Soykoek S, Mall V, Nehring I (2017). Post-traumatic stress disorder in Syrian children of a German refugee camp. Lancet.

[6] Human Rights (1989) Convention on the rights of the child. https://www.unicef.org/sites/default/files/2019-04/UN-Convention-Rights-Child-text.pdf. Accessed 23 Nov 2020

[7] Mickley M, Renner G (2019). Auswahl, Anwendung und Interpretation deutschsprachiger Intelligenztests für Kinder und Jugendliche auf Grundlage der CHC-Theorie: Update, Erweiterung und kritische Bewertung. Prax Kinderpsychol Kinderpsychiatr.

[8] Manly JJ (2008). Critical issues in cultural neuropsychology: profit from diversity. Neuropsychol Rev.

[9] Roth B, Becker N, Romeyke S (2015). Intelligence and school grades: a meta-analysis. Intelligence.

[10] Lynn R, Vanhanen T (2012). National IQs: a review of their educational, cognitive, economic, political, demographic, sociological, epidemiological, geographic and climatic correlates. Intelligence.

[11] Trahan LH, Stuebing KK, Fletcher JM, Hiscock M (2014). The Flynn effect: a meta-analysis. Psychol Bull.

[12] Hagmann-von Arx P, Petermann F, Grob A (2013). Konvergente und diskriminante Validität der WISC- IV und der Intelligence and Development Scales (IDS) bei Kindern mit Migrationshintergrund. Diagnostica.

[13] Schölmerich A, Leyendecker B, Citlak B (2008). Assessment of migrant and minority children. Zeitschrift für Psychologie / Journal of Psychology.

[14] Lüdeke S, Linderkamp F, Weidenfeld A, Borusiak P (2015). Die Einflüsse des Migrationshintergrundes und psychischer Störungen auf kognitive Leistungsdaten einer SPZ-Inanspruchnahmepopulation. Kindheit und Entwicklung.

[15] Kuhlman KR, Chiang JJ, Horn S, Bower JE (2017). Developmental psychoneuroendocrine and psychoneuroimmune pathways from childhood adversity to disease. J Neubiorev.

[16] Finegood ED, Wyman C, O’Connor TG (2017). Salivary cortisol and cognitive development in infants from low-income communities. Stress.

[17] Suor JH, Sturge-Apple ML, Davies PT, Cicchetti D, Manning LG (2015) Tracing Differential Pathways of Risk: Associations Among Family Adversity, Cortisol, and Cognitive Functioning in Childhood. Child Dev 86(4):1142–1158. 10.1111/cdev.12376 10.1111/cdev.12376 PMC4683120 26081792

[18] Melchers P, Melchers M (2015) Kaufman-Assessment-Battery for Children (KABC-II) by Kaufman AS, Kaufman NL. German version. Frankfurt/Main, Pearson

[19] Irblich D, Schroeder A, Renner G (2020). Psychometrische Eigenschaften der “Kaufman Assessment Battery for Children - II” (KABC-II) bei 5- und 6-jährigen Kindern. Frühförderung interdisziplinär.

[20] Hahnefeld A, Sukale T, Weigand E, Münch K, Aberl S, Eckler L, Schmidt D, Friedmann A, Plener P, Fegert J, Mall V (2021). Survival states as indicators of learning performance and biological stress in refugee children: a cross-sectional study with a comparison group. BMC Psychiatry.

[21] Sukale T, Hertel C, Möhler E (2017). Diagnostik und Einschätzung bei minderjährigen Flüchtlingen. Nervenarzt.

[22] Luria A (1970) The functional organization of the brain. Scientific American 222(3):66–79. Retrieved November 11, 2020, from http://www.jstor.org/stable/24925755 10.1038/scientificamerican0370-66 5413155

[23] Carroll JB, Flanagan DP, Genshaft JL, Harrison PL (1997). The three-stratum theory of cognitive abilities. Contemporary intellectual assessment: Theories, tests, and issues.

[24] Kuschel A, Kamp-Becker I & Ständer D (2017) TBS-TK Rezension: »Kaufman Assessment Battery for Children-2 (KABC-II)«. report psychologie 5:211

[25] Fletcher-Janzen E, Ortiz S (2006). Cultural competence in the use of IQ tests with culturally and linguistically diverse children. Gift Educ Int.

[26] Pathways to Wellness. Integrating refugee health and well-being. Pathways to Wellness.http://refugeehealthta.org/wp-content/uploads/2012/09/RHS15_Packet_PathwaysToWellness-1.pdf. Published 2011. Accessed 8 May 2020

[27] Sachser C, Berliner L, Holt T (2017). International development and psychometric properties of the Child and Adolescent Trauma Screen (CATS). J Affect Disord.

[28] American Psychiatric Association (2013) Diagnostic and statistical manual of mental disorders. 5th ed. Arlington: American Psychiatric Association

[29] Lenhard W, Lenhard A (2017) Computation of effect sizes. 10.13140/RG.2.2.17823.92329

[30] Dilling H, Mombour W, Schmidt M, Schulte-Markwort E, Remschmidt H, WHO (2015) Internationale Klassifikation psychischer Störungen. 10th ed. Hogrefe, Bern

[31] Fujii D (2018). Developing a cultural context for conducting a neuropsychological evaluation with a culturally diverse client: the ECLECTIC framework. Clin Neuropsychol.

[32] Rosselli M, Ardila A (2003). The impact of culture and education on non-verbal neuropsychological measurements: a critical review. Brain Cogn.

[33] Voigt F (2018). Kaufman Assessment Battery for Children-II (KABC-II): Aufbau des Verfahrens und Erfahrungen mit der Anwendung im Vorschulbereich. Frühförderung interdisziplinär.

[34] Greenfield P (1997). You can’t take it with you: why ability assessments don’t cross cultures. Am Psychol.

[35] Casaletto KB, Heaton RK (2017). Neuropsychological assessment: past and future. J Int Neuropsychol Soc.

